# A Practical Treatise on the Diseases of Children

**Published:** 1850-01

**Authors:** 


A PRACTICAL TREATISE ON THE DISEASES OF CHILDREN, BY D. FRANCIS CONDIE, M. D. THIRD EDITION, REVISED AND AUGMENTED. LEA & BLANCHARD. 1850.

We are glad to see that the merits of this valuable work which we noticed at length on the publication of the first edition, have been so correctly appreciated, that a new edition, revised and augmented, has been prepared to meet the demand. The work is one of much ability, and is worthy of the confidence of the profession, and as such we commend it to their attention.

